# Prevalence of Anemia Among Adolescent Girls in Sub-Saharan Africa: Systematic Review and Meta-Analysis

**DOI:** 10.3389/phrs.2025.1608303

**Published:** 2025-11-12

**Authors:** Mequanente Dagnaw, Daniel Gashaneh Belay, Tsega Degu, Dessie Abebaw Angaw, Tsheten Tsheten

**Affiliations:** 1 Department of Epidemiology and Biostatistics, Institute of Public Health, College of Medicine and Health Sciences, University of Gondar, Gondar, Ethiopia; 2 Department of Medical Biotechnology, Institute of Biotechnology, University of Gondar, Gondar, Ethiopia; 3 National Centre for Epidemiology and Population Health, Australian National University, Acton ACT 2601, Canberra, Australia

**Keywords:** anemia, adolescent girl, systematic review, meta-analysis, Sub-Saharan Africa

## Abstract

**Objectives:**

To estimate the prevalence of anemia among adolescent girls in Sub-Saharan Africa through a systematic review and meta-analysis.

**Methods:**

A systematic review and meta-analysis of both published and unpublished studies conducted in Sub-Saharan Africa were employed in this study. Using pertinent search terms, all research found in Google Scholar, HINARI, Cochrane Library, and PubMed was located. Following the evaluation of the evidence with the Joanna Briggs Institute methodology for cross-sectional studies, data were extracted.

**Results:**

Thirty studies met the inclusion criteria, comprising a total sample of 12,295 adolescent girls. The pooled prevalence of anemia among adolescent girls in Sub-Saharan Africa was 30% [95% CI: 29%–32%], with significant heterogeneity observed (I^2^ = 99.2%, p < 0.001).

**Conclusion:**

The high pooled prevalence of anemia among adolescent girls in Sub-Saharan Africa indicates a substantial public health concern. This review provides up-to-date epidemiological data that can support health policymakers. Strengthening anemia prevention and control strategies, along with establishing targeted interventions at local health system levels, is crucial.

## Introduction

Adolescence, defined by the World Health Organization (WHO) as the period between 10 and 19 years of age, is characterized by rapid physical, psychological, and cognitive development [1]. During this transitional phase, particularly with the onset of menstruation and the adolescent growth spurt, iron requirements significantly increase. As a result, adolescent girls are at heightened risk of iron deficiency and anemia [2, 3].

Anemia, a condition in which hemoglobin concentration or red blood cell count falls below the standard threshold, impairs the blood’s capacity to transport oxygen [4]. Globally, anemia remains a critical public health concern, affecting over two billion people approximately a quarter of the world’s population [5]. Over two billion people, or roughly a quarter of the world’s population, are thought to have anemia, according to the World Health Organization (WHO) [6]. It disproportionately affects women of reproductive age, young children, and adolescent girls, especially in low- and middle-income region [6].

In sub-Saharan Africa (SSA), anemia in adolescent girls is driven primarily by nutritional deficiencies, parasitic infections such as malaria and hookworm, and limited access to healthcare and nutrition services. Nutritional deficiencies are the most common cause of anemia worldwide, and in sub-Saharan Africa, they constitute a significant risk factor for anemia in teenage girls [3, 7]. In refugee settings, where food insecurity and inadequate sanitation are common, adolescent girls face heightened vulnerability, with anemia prevalence reaching up to 69.2% as reported in the Fugnido Refugee Camp in Ethiopia [2, 3].

While multiple studies have investigated anemia prevalence and its determinants among adolescent girls in SSA, the results are inconsistent and fragmented, and to date, few systematic reviews or meta-analyses have specifically targeted this population in the region [7, 8]. Similarly, 45% of Iranian women who had been refugees for at least 10 years were anemic [8, 9]. In the Fugnido Refugee Camp in Gambella, Western Ethiopia, 69.2% of refugee females aged 10 to 19 who received food help had anemia [3, 10]. This presents a gap in evidence synthesis that could help shape regional and country-level nutrition and health policies.

The consequences of anemia during adolescence are far-reaching. It compromises physical growth, academic performance, and future reproductive outcomes [11]. When anemia persists into early pregnancy, it increases the risk of maternal mortality, adverse birth outcomes, and long-term developmental issues in infants [3, 12]. Pregnancy during adolescent with anemia raises maternal morbidity and mortality rates as well as the likelihood of unfavorable pregnancy outcomes like stillbirth, low birth weight, and premature birth. It also has a detrimental effect on the infant’s iron status [11, 12].

Anemia in adolescent girls is caused by a variety of circumstances, according to several academic studies. Anemia was substantially correlated with low dietary diversity, long menstrual cycles, a history of malaria attacks, large families and low elevations, household food insecurity, and low dietary diversity [11, 13]. Given the significance of anemia particularly for adolescent girls, who are an endangered population and the considerable variation in its reported prevalence, a systematic examination of the literature is required to gain a deeper understanding of the various facets of this issue in Ethiopian society. Through a systematic review and meta-analysis of relevant data, the current study aims to provide a thorough evaluation of the prevalence level of anemia among teenage girls. Policymakers would benefit from having a more accurate plan and a more realistic perspective on the situation as they work to lessen the burden of this condition. The purpose of this review is to ascertain the prevalence of anemia and the factors that contribute to it in teenage females in Sub-Saharan African countries.

Despite the high burden of anemia in Sub-Saharan Africa there is only few evidences with different magnitude. The number of new publications published in health journals is growing exponentially. There is a growing focus on the need for healthcare decisions and policies to be based on the best available evidence. Variability between studies (effect size, outcome measurement, and sample size and study year) marked variations in the prevalence and factors of anemia were noted among adolescent girls between studies in Sub-Saharan Africa. To work on modifiable risk factors and improve the level of treatment outcome knowing the magnitude and associated factors is very vital. Nutritional consumption there are not many findings with varying magnitudes, despite the significant prevalence of anemia in Sub-Saharan Africa. The magnitude reflected in these fragmented studies varied widely and remained inconclusive. Pooling estimates at Sub-Saharan Africa level would provide precise findings & it may contribute to inform policymakers to design interventions, i.e., allocate resources and services to anemia patients. So, it’s crucial to search and understand evidences about anemia in adolescents and estimate the pooled prevalence.

Given those concerns, the current systematic review and meta-analysis (SRMA) aims to estimate the pooled prevalence of anemia and its associated factors among adolescent girls in sub-Saharan Africa. Understanding the burden and determinants of anemia in this population will aid policymakers in developing targeted, evidence-based strategies to reduce its prevalence and improve adolescent health outcomes in the region.

## Methods

### Study Design and Review Framework

This systematic review and meta-analysis aimed to estimate the pooled prevalence of anemia and identify its associated factors among adolescent girls in sub-Saharan Africa, following the PRISMA 2020 guidelines. Although we intended to register the protocol with PROSPERO, this was not feasible due to the platform’s prioritization of COVID-19-related submissions at the time. The inclusion and exclusion criteria were developed based on the PICO framework: Population adolescent girls aged 10–19 years in sub-Saharan Africa; Intervention/Exposure risk or protective factors for anemia such as infections, dietary diversity, or iron and folic acid supplementation; Comparator adolescents without these exposures or with varying levels of exposure; Outcome prevalence or odds of anemia. Only quantitative cross-sectional observational studies published in English and reporting original primary data were included, while qualitative studies, reviews, editorials, and studies lacking original prevalence data were excluded.

### Literature Search Strategy

A systematic literature search was conducted across PubMed, Medline, and Google Scholar databases for relevant studies published up to February 25, 2020. The search used a combination of Boolean operators and keywords, including “Anemia” AND (“adolescent*” OR “teenager” OR “youth”) AND (“girl” OR “woman”), along with terms such as “associated factors” or “determinants.” Additionally, the names of all sub-Saharan African countries (e.g., Kenya, Ethiopia, Nigeria, Ghana, Uganda, etc.) were included in the search to capture country-specific evidence. Reference lists of eligible articles were also screened to identify any additional studies. Only studies published in English were included.

### Selection of the Studies

We included observational studies reporting the prevalence and/or associated factors of anemia among adolescent girls in sub-Saharan Africa. Studies published in any year up to the cut-off date and employing a quantitative cross-sectional design were considered. Qualitative studies and those not reporting primary prevalence data were excluded. Two independent reviewers screened titles, abstracts, and full texts according to predefined eligibility criteria. Disagreements were resolved by discussion or a third reviewer.

### Data Extraction and Quality Assessment

A standardized and pilot-tested data extraction form was used to collect relevant information from each included study. Extracted variables included the first author, year of publication, study country, study setting (community- or facility-based), sample size, anemia prevalence, and associated factors such as odds ratios (ORs), confidence intervals (CIs), and p-values. Data extraction was performed independently by two reviewers, and the results were cross-checked to ensure accuracy. Any discrepancies between reviewers were resolved through discussion and consensus. In cases where critical data were missing or unclear, study authors were contacted via email for clarification. The quality of the studies was evaluated in terms of inclusion using the Modified Newcastle Ottawa quality assessment scale for cross-sectional studies. Cross-sectional studies were graded using the modified Newcastle-Ottawa scale, which had a maximum overall score of nine [9] stars and a minimum of zero. A study was deemed to be of high quality if it received seven out of nine stars, and medium if it received five out of nine [14]. The woman’s anemia during pregnancy was the study’s main finding. Variables like age, family size, wealth, and parity were sought after, however published studies did not yield enough data. Intestinal parasite, iron and folic acid supplements, third trimester pregnancy, and Dietary Diversified Score (DDS) are a few potential causes of anemia. Thus, the 24-hour dietary recall method was used to gather data on nutrient intake in order to calculate the dietary diversity score. The result is divided into three categories: low (DDS 3), medium (DDS = 4 or 5), and high (DDS >5). The fourth antenatal visit, which is defined as adolescent females who had antenatal care four or more times throughout the pregnancy period, is the last point of clarification (Table 1).

**TABLE 1:** Data extraction for systematic review and meta-analysis of prevalence of anemia among adolescent girls in Sub-Saharan Africa (Amhara, Ethiopia, 2020).

Authors	Year	Countries	Study design	Sample size	Anemia definition
Mengistu G et al.	2018	Ethiopia	ACS	443	Hemoglobin levels less than 12.5 g/dL
Tura et al.	2020	Ethiopia	ACS	523	Hemoglobin levels less than 12.5 g/dL
Engdaw M et al. [8]	2018	Ethiopia	ACS	437	Hemoglobin levels less than 12.5 g/dL
Fentie et al.	2020	Ethiopia	ACS	528	Hemoglobin levels less than 12.5 g/dL
Gebreyesus et al.	2019	Ethiopia	ACS	384	Hemoglobin levels less than 12.5 g/dL
Gutie et al.	2018	Ethiopia	ACS	462	Hemoglobin levels less than 12.5 g/dL
Gosdin et al.	2020	Ghana	DCS	2948	Hemoglobin levels less than 12.5 g/dL
Gutie et al.	2019	Ethiopia	ACS	400	Hemoglobin levels less than 12.5 g/dL
Leenstra et al.	2004	Kenya	ACS	633	Hemoglobin levels less than 12.5 g/dL
Nelima	2015	Kenya	ACS	230	Hemoglobin levels less than 12.5 g/dL
Regassa et al.	2019	Ethiopia	ACS	454	Hemoglobin levels less than 12.5 g/dL
Tandoh et al.	2021	Ghana	DCS	151	Hemoglobin levels less than 12.5 g/dL
Teni et al.	2017	Ethiopia	ACS	442	Hemoglobin levels less than 12.5 g/dL
Zeleke et al.	2020	Ethiopia	DCS	365	Hemoglobin levels less than 12.5 g/dL
Adelman et al.	2019	Uganda	ACS	65	Hemoglobin levels less than 12.5 g/dL
Abdelrahim	2009	Sudan	DCS	187	Hemoglobin levels less than 12.5 g/dL
Agyeponng	1997	Ghana	DCS	112	Hemoglobin levels less than 12.5 g/dL
Agyeponng	1997	Ghana	DCS	101	Hemoglobin levels less than 12.5 g/dL
Alaofe et al.	2009	Benin	ACS	34	Hemoglobin levels less than 12.5 g/dL
Alaofe et al.	2007	Benin	ACS	100	Hemoglobin levels less than 12.5 g/dL
Barr et al.	1998	Nigeria	DCS	309	Hemoglobin levels less than 12.5 g/dL
Demelash et al.	2015	Ethiopia	ACS	594	Hemoglobin levels less than 12.5 g/dL
Massawe et al.	2002	Tanzania	ACS	130	Hemoglobin levels less than 12.5 g/dL
A Woodruff et al.	2005	Kenya	DCS	388	Hemoglobin levels less than 12.5 g/dL
Adem et al.	2015	Ethiopia	ACS	338	Hemoglobin levels less than 12.5 g/dL
Shaka et al.	2018	Ethiopia	ACS	177	Hemoglobin levels less than 12.5 g/dL
Tesaye et al.	2015	Ethiopia	ACS	232	Hemoglobin levels less than 12.5 g/dL
Alemu et al.	2019	Ethiopia	ACS	406	Hemoglobin levels less than 12.5 g/dL
Teji et al.	2016	Ethiopia	ACS	547	Hemoglobin levels less than 12.5 g/dL
Ayogu et al.	2016	Nigeria	ACS	175	Hemoglobin levels less than 12.5 g/dL

### Statistically Analysis

Extracted data were initially entered into Microsoft Excel and then imported into Review Manager (RevMan 5.3) and Comprehensive Meta-Analysis (CMA v2) software for statistical analysis. Due to expected differences across studies in populations and methods, a random-effects model was selected to account for both within-study and between-study variability. Pooled estimates were calculated for anemia prevalence as well as odds ratios (ORs) of key risk factors, such as intestinal parasite infection, dietary diversity, and supplementation. For pooling ORs, a minimum of three studies reporting the same variable was required.

Heterogeneity among studies was assessed using the I^2^ statistic, with values of 25%, 50%, and 75% representing low, moderate, and high heterogeneity, respectively. Subgroup analyses were conducted based on geographical sub-regions (East, West, and Central Africa), study setting (community versus facility-based), and study quality (high versus moderate). To assess the robustness of the findings, sensitivity analysis was performed, resulting in the exclusion of five outlier studies that substantially influenced the pooled results.

Publication bias was evaluated through both graphical and statistical methods. Funnel plots were visually inspected for asymmetry, and Egger’s regression test was conducted to detect potential bias. To further adjust for the possible effects of unpublished studies, the trim-and-fill method was applied to correct the pooled estimates.

### Ethical Consideration

Ethical approval was not required for this review as all data were extracted from previously published studies.

## Results

### Studies Selection

**FIGURE 1:**
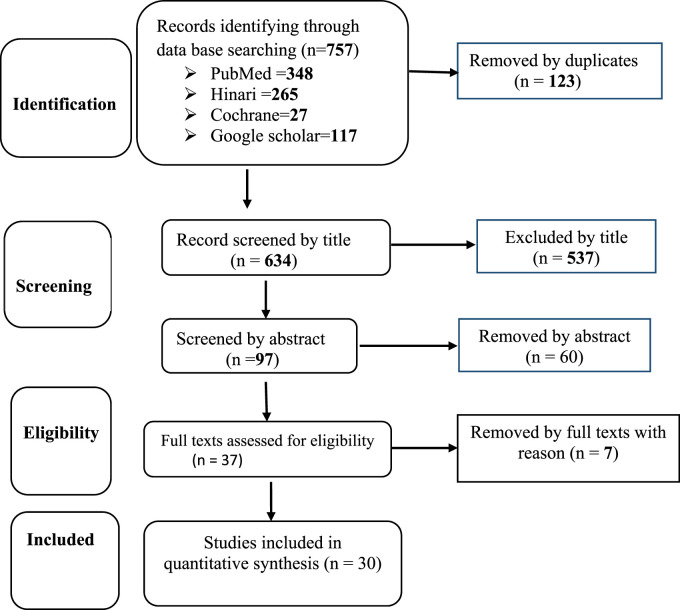
Flow diagram of the included studies for the systematic review and meta-analysis of prevalence of anemia among adolescent girl in sub-Saharan countries (Amhara, Ethiopia, 2020).

Before delving to the details of the results, we identified several papers related to the prevalence and determinants of anemia for the inclusion in meta-analysis based on the goals outlined for this study. In total, we identified 757 studies that were published in international journals. Of these, 736 were excluded because they did not meet the inclusion criteria established for this review. Ultimately Only 30 studies were included in this analysis due to their rigorous design and comprehensive explanations (Figure 1).

### Characteristics of Included Studies

The meta-analysis included thirty cross-sectional studies conducted across various African countries. Among these, fifteen studies (50%) were from Ethiopia, four (13%) from Ghana, three (10%) from Kenya, one (3%) from Uganda, one (3%) from Tanzania, three (10%) from Nigeria, one (3%) from Sudan, and two (6%) from Benin. The study conducted in Ghana [15] had the largest sample size, totaling 2,948 participants, while the smallest sample size was found in Benin, with only 34 participants.

### Prevalence of Anemia

**FIGURE 2:**
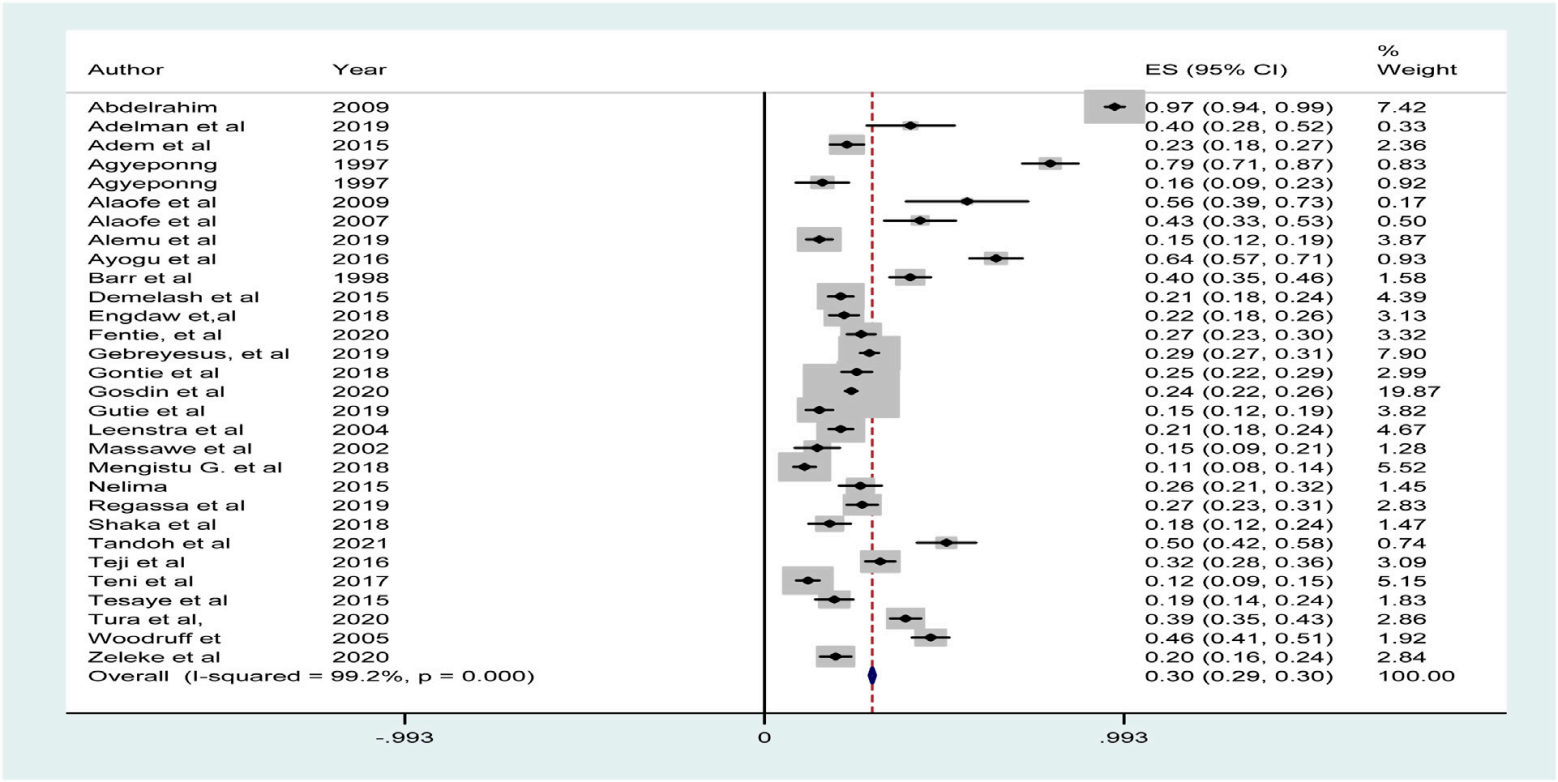
The pooled prevalence of anemia among adolescent girls’ women in Sub-Saharan Africa (Amhara, Ethiopia, 2020).

The analysis revealed that Ethiopia had the lowest prevalence of anemia (11.1%) among the included studies. In contrast, the study conducted in Sudan showed a strikingly high prevalence of 96.8%. The pooled estimate for the prevalence of anemia among adolescent girls in sub-Saharan Africa was 30% [95% CI = 0.29–0.30], with an I^2^ value of 99.2% (p-value = 0.00) (Figure 2).

### Meta-Analysis for Proportion (Random)

**FIGURE 3:**
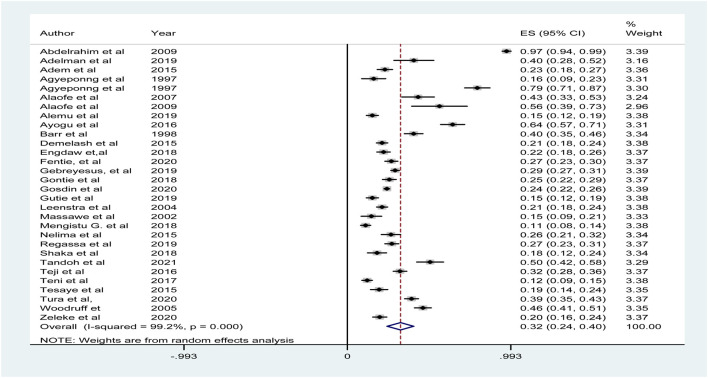
Displaying the proportion (random) prevalence of anemia among adolescent girls in Sub-Saharan Africa (Amhara, Ethiopia, 2020).

Since the I-square fixed effect has shown heterogeneity, random pooled estimate of the anemia among adolescent girl was performed and the result shows 32% [24–40] with I^2^ = 99.2% (p- value = 0.00) (Figure 3).

### Subgroup Analysis

To further explore sources of heterogeneity, subgroup analyses were conducted based on study setting, geographical region within sub-Saharan Africa, and year of publication. The decision to use regional rather than national groupings was driven by the limited number of studies per country, which precluded reliable country-level estimates. Regional subgrouping allowed for better statistical power and meaningful comparison. The prevalence estimates and corresponding 95% confidence intervals (CI) for each subgroup are see in (Supplementary Table S1).

### Sensitivity Analysis

To assess the robustness of our findings and identify influential studies, we performed a leave-one-out sensitivity analysis. Five studies were found to be outliers, with extreme prevalence values that significantly inflated the overall heterogeneity (I^2^). These studies had prevalence rates that were either exceptionally low or high and were excluded from the final pooled analysis due to their disproportionate influence on the effect estimate.

**FIGURE 4:**
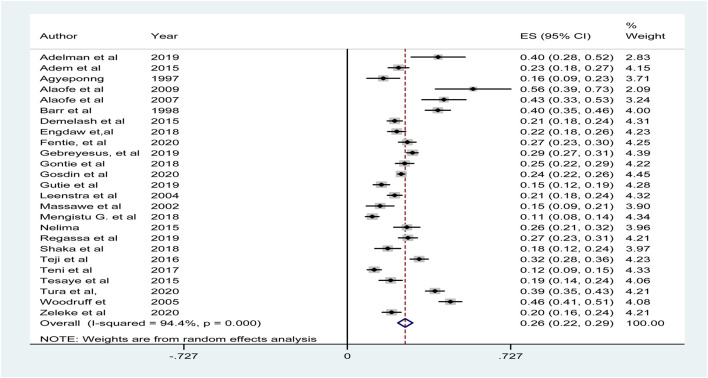
The result of Sensitivity analysis after drop of five variables prevalence of anemia among adolescent girls in Sub-Saharan Africa (Amhara, Ethiopia, 2020).

After removing these five studies, the recalculated pooled prevalence was 26% and heterogeneity was reduced to I^2^ = 94.4% (p = 0.00). This adjustment demonstrates the stability of the pooled result (Supplementary Figure S1; Figure 4).

### Meta Regression

Since trim and fill approaches are resilient for estimation due to publication bias, they were employed to estimate the pooled prevalence anemia and the effect size of factors associated with anemia in teenage girls in the context of the small-study effect. The measure of association between anemia and related factors was estimated using the odds ratio and its 95% confidence interval (Supplementary Table S2).

### Publication Bias (Small Study Biases)

**FIGURE 5:**
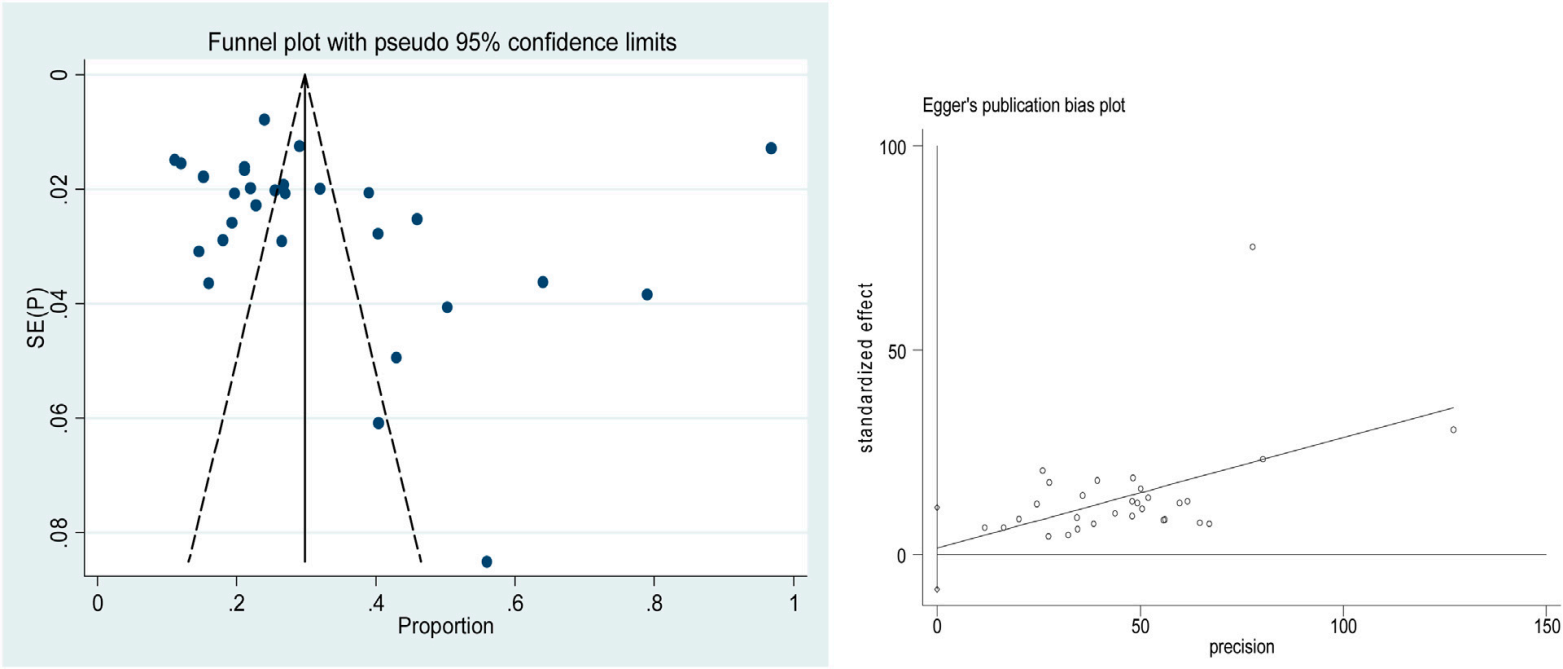
A graphical inspection of publication bias and publication bias treatment using a funnel plot, trim and fill assessment of effect sizes versus the standard error of the effect sizes of selected studies (Amhara, Ethiopia, 2020).

The egger test result showed that there were no publication biases since the p-value of the bias is not statistically significant (p = 0.75). Even if the Meta funnel graph is subjective to show the small study bias, Figure 5 showed that the standard error is distributed symmetrical with proportion (Figure 5).

## Discussion

Anemia remains a persistent public health issue in Sub-Saharan Africa (SSA), particularly affecting adolescent girls who are undergoing rapid physical growth and, in many cases, beginning menstruation both of which increase iron requirements [16]. Our systematic review and meta-analysis, which pooled data from 30 cross-sectional studies involving 11,895 participants, found a pooled anemia prevalence of 30% (95% CI: 29%–0.32%) among adolescent girls in SSA. This aligns with the World Health Organization’s classification of anemia as a moderate public health problem in this context.

Globally, approximately 38% (32 million) of adolescent girls’ experience anemia, but prevalence rates vary widely. For example, in high-income countries like Iran, prevalence is around 10% [17], highlighting stark contrasts driven by socioeconomic factors, dietary practices, and healthcare infrastructure. In sub-Saharan Africa, anemia is more common than in developed nations. In SSA, the prevalence ranged from as low as 11.1% (Ethiopia) to as high as 96.8% (Sudan), indicating considerable heterogeneity across regions and study settings. Our subgroup analyses attributed much of this variability to differences in geographic location (e.g., East vs. West Africa) and study context (community-based vs. refugee settings [18].

Notably, the pooled prevalence among adolescent girls in Ethiopia was also 30%, mirroring the regional average. However, this figure still indicates that roughly one in three adolescent girls is affected. In many cases, this means that girls receiving services in schools, rural towns, or even humanitarian settings are not reaching adequate iron levels needed for optimal health and development.

Cultural and behavioral factors may further exacerbate the burden of anemia. Limited dietary diversity, food taboos, lack of awareness about nutritional needs, and gender-based disparities in food access all contribute to inadequate iron intake. Additionally, in some settings, heavy or irregular menstrual cycles may go unaddressed due to stigma or lack of access to healthcare, compounding the risk.

While several countries in SSA have adopted iron supplementation and school-based nutrition programs, coverage is inconsistent, and implementation challenges persist especially in remote or underserved regions. Our findings underline the urgent need to strengthen and expand current interventions, improve dietary education, and integrate anemia prevention into broader adolescent health initiatives.

### Strength and Limitation

This systematic review and meta-analysis followed a rigorous methodological process, with multiple reviewers involved at every stage. To ensure transparency and reproducibility, we adhered to the PRISMA (Preferred Reporting Items for Systematic Reviews and Meta-Analyses) guidelines throughout.

Despite these strengths, several limitations should be acknowledged. First, there was a lack of data from several countries in sub-Saharan Africa, and in some regions, only a small number of studies were available, limiting generalizability. Second, the pooled prevalence estimates may have been affected by methodological differences among included studies, particularly variability in screening techniques and diagnostic criteria. These inconsistencies contribute to the observed heterogeneity across studies.

Additionally, regional disparities in healthcare access, nutritional status, and socio-economic conditions likely influence the reported prevalence, yet were not uniformly accounted for. The reliance on cross-sectional studies with differing sample sizes and designs further introduces variability. These limitations suggest that while the findings provide a useful overview, the pooled prevalence estimate should be interpreted with caution.

Future studies should aim to improve regional representation, adopt standardized screening protocols, and explore the contextual factors contributing to anemia prevalence. This will help produce more robust and actionable evidence to inform public health strategies.

## Conclusion

This meta-analysis demonstrates that anemia among adolescent girls in sub-Saharan Africa remains a significant public health issue, with a pooled prevalence of approximately 30%. This figure underscores the persistent burden of anemia in the region, especially when compared to lower prevalence rates reported in high-income countries.

By synthesizing data from a range of recent studies, this review contributes to a clearer understanding of the regional burden and highlights opportunities for targeted interventions. However, the variability in study quality, methodologies, and geographic coverage must be considered when interpreting the findings.

To address this burden, multi-faceted, context-specific strategies are needed. These may include school-based screening programs, improved iron and folic acid supplementation, and culturally tailored community education initiatives. The “screen-and-treat” approach could also be explored in settings where resources allow.

Further research is needed, particularly in underrepresented countries, and should aim to disaggregate findings by age within adolescence to better inform age-appropriate interventions. National health policies should prioritize adolescent anemia prevention as part of broader health and education system strengthening. Expanding the geographic scope of anemia control programs, standardizing screening methodologies, and enhancing political commitment and public awareness are essential for effective long-term solutions.
