# Supplementary material for: Prevalence of Anemia Among Adolescent Girls in Sub-Saharan Africa: Systematic Review and Meta-Analysis
Source: Public Health Rev. 2025 Nov 12;46:1608303. doi: 10.3389/phrs.2025.1608303 (PMC12646963; doi:10.3389/phrs.2025.1608303)
Supplement: Supplementary file 1 [file Supplementaryfile1.docx]

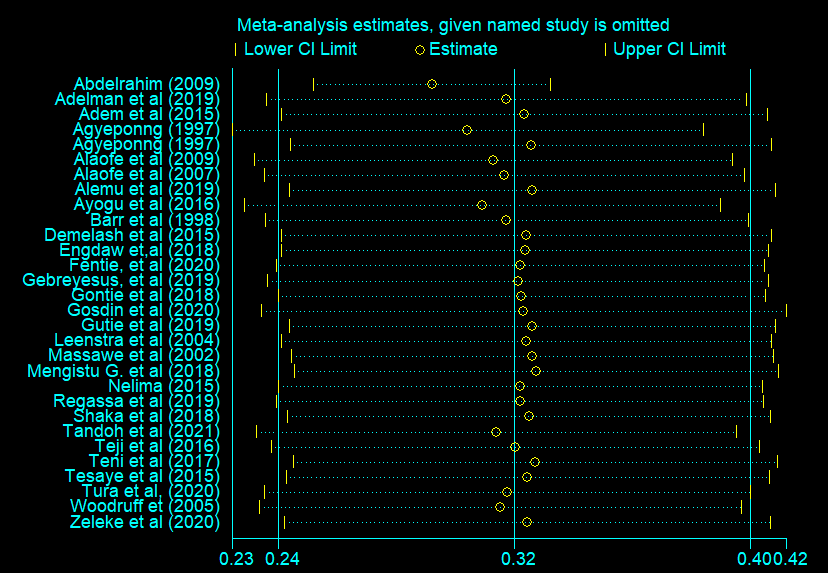


**Supplementary Figure 1.** Sensitivity analysis before omitted studies prevalence of anemia among adolescent girls in Sub-Saharan Africa
