# Supplementary material for: Prevalence of Anemia Among Adolescent Girls in Sub-Saharan Africa: Systematic Review and Meta-Analysis
Source: Public Health Rev. 2025 Nov 12;46:1608303. doi: 10.3389/phrs.2025.1608303 (PMC12646963; doi:10.3389/phrs.2025.1608303)
Supplement: Supplementary file 2 [file Table1.docx]

**Supplementary Table 1.** Subgroup analysis of prevalence of anemia among adolescent girls in Sub-Saharan Africa

| **Subgroup** | **No. of included studies** | **Prevalence(95%CI)** | **P value** | **I^2^** |
| --- | --- | --- | --- | --- |
| **Study setting** | School | 33%(21,44) | 0.00 | 99.4% |
|  | Community | 31%(22,44) | 0.00 | 97.3% |
|  | Refugee | 34%(10,57) | 0.00 | 98.2% |
| **Region** | East Africa | 27%(17,37) | 0.00 | 99.4% |
|  | West Africa | 44%(25,63) | 0.00 | 98.1% |
|  | North Africa | 52%(29,75) | 0.00 | 96.3% |
| **Year of Publications** | >2015 year | 27%(23,32) | 0.00 | 96.5% |
|  | <2015 year | 39%(20,58) | 0.00 | 99.5 |
