# Supplementary material for: Prevalence of Anemia Among Adolescent Girls in Sub-Saharan Africa: Systematic Review and Meta-Analysis
Source: Public Health Rev. 2025 Nov 12;46:1608303. doi: 10.3389/phrs.2025.1608303 (PMC12646963; doi:10.3389/phrs.2025.1608303)
Supplement: Supplementary file 3 [file Table2.docx]

**Supplementary Table 2**. Multivariate Meta regression analysis of anemia among adolescent girls in Sub-Saharan Africa.

| **Variable name** | **Multivariate meta regression** | | | |
| --- | --- | --- | --- | --- |
|  | Coef. (95%) | P-value | I^2^ | Adjusted R^2^ |
| **Sample size** | -0.00009 | 0.186 | 98.4 | 20.67 |
| **Years** | -0.06449 | 0.375 | 98.4 | 20.67 |
| **Region** | 0.113198 | 0.062 | 98.4 | 20.67 |
| **Design** | 0.11479 | 0.117 | 98.4 | 20.67 |
